# Rev-erbα inhibits proliferation by reducing glycolytic flux and pentose phosphate pathway in human gastric cancer cells

**DOI:** 10.1038/s41389-019-0168-5

**Published:** 2019-10-07

**Authors:** Linlin Tao, Haoyuan Yu, Rui Liang, Ru Jia, Jingjing Wang, Kai Jiang, Zhengguang Wang

**Affiliations:** 1 Department of General Surgery, The First Affiliated Hospital of Anhui Medical University, Hefei, Anhui People’s Republic of China; 2 Division of Infectious Diseases, Department of Infectious Diseases, The First Affiliated Hospital of University of Science and Technology of China, Hefei, Anhui People’s Republic of China

**Keywords:** Cancer metabolism, Cell growth

## Abstract

Rev-erbα is a nuclear receptor, which regulates circadian rhythm, inflammatory responses and lipid metabolism. We previously showed Rev-erbα reduction in human gastric cancer, which is associated with TMN stages and poor prognosis. We hypothesized that Rev-erbα modulates proliferation via glycolytic flux and the pentose phosphate pathway (PPP) in gastric cancer. Knockdown of Rev-erbα significantly increased proliferation as well as glycolytic flux and the PPP in human gastric cancer cells. These effects were reduced by a Rev-erbα agonist GSK4112 in a dose-dependent manner. Furthermore, Rev-erbα was recruited on the promoters of PFKFB3 and G6PD genes, thereby inhibiting their gene transcription. GSK4112 treatment reduced PFKFB3 and G6PD gene expression, which was not affected by BMAL1 knockdown. Pharmacological inhibition of glycolysis and the PPP using corresponding PFKFB3 and G6PD inhibitors attenuated Rev-erbα knockdown-induced proliferation in gastric cancer cells. GSK4112 treatment was not able to reduce proliferation in SGC-7901 overexpressing both PFKFB3 and G6PD genes. Both PFKFB3 and G6PD were overexpressed in patients with gastric cancer, and positively correlated with the TMN stages. The PPP and glycolysis were enhanced in gastric cancer tissues of patients with low expression of Rev-erbα compared to the patients with high expression of Rev-erbα. In conclusion, Rev-erbα reduction causes gastric cancer progression by augmenting the PPP and glycolysis.

## Introduction

Gastric cancer is the second most common cause of cancer-related death worldwide^1,2^. Gastric cancer cells rely on aerobic glycolysis instead of oxidative phosphorylation providing bioenergetics and macromolecule synthesis for their proliferation, which is termed ‘Warburg effect’. It has been shown that the levels and/or enzymatic activities of hexokinase II (HKII), phosphosphofructokinase (PFK), M2 type of pyruvate kinase (PKM2), 6-phosphofructokinase-2/fructose-2,6-bisphosphatase (PFKFB3), and glucose-6-phosphate dehydrogenase (G6PD) involved in glycolysis and pentose phosphate pathway are increased, which results in the progression and/or poor prognosis of gastric cancer^3–10^. In fact, reoperative metabolic syndrome is predictive of significant gastric cancer mortality after gastrectomy^11^. However, the mechanisms underlying these increased enzymes in gastric cancer are not clear.

Rev-erb is a nuclear receptor, which has two members, Rev-erbα and Rev-erbβ, and serves as receptor for heme. Rev-erb plays a number of diverse and important roles in regulation of circadian rhythm, inflammatory responses, senescence, and lipid in central nervous system and peripheral organs^12,13^. There are limited studies regarding the role of Rev-erb in tumors. Rev-erbα mRNA levels are significantly reduced in breast cancer cells, while Rev-erbβ mRNA expression is significantly increased^14^. Activation of Rev-erbα/β inhibits breast cancer cell proliferation, and is cytotoxic in cancer cells derived from different tumor types, namely brain, leukemia, breast, colon and melanoma^13,15^. Our recent study showed that the expression of Rev-erbα was decreased in human gastric cancer, which was associated with TMN stages and poor prognosis^16^. However, it is unknown whether Rev-erbα modulates glycolysis and subsequent proliferation in gastric cancer cells. We, therefore, hypothesize that Rev-erbα inhibits glycolysis and pentose phosphate pathway, thereby reduces proliferation in gastric cancer cells.

## Materials and methods

### Cell cultures and treatments

The human gastric cancer cell lines, including SGC-7901, BGC-823, and AGS, were purchased from the American Type Culture Collection (ATCC, Manassas, VA, USA). There was no mycoplasma contamination. Cells were cultured in Dulbecco’s modified Eagle’s medium (DMEM; Gibco; Thermo Fisher Scientifc, Inc., Waltham, MA, USA) containing high glucose, L-glutamine, pyridoxine hydrochloride, sodium pyruvate and bicarbonate along with 10% heat inactivated fetal bovine serum (Clark Bioscience, Richmond, VA, USA), 100 U/ml penicillin and 100 µg/ml streptomycin at 37 °C with 5% CO_2_ in a humidified atmosphere. Cells were treated with GSK4112 (0.5 μM and 2 μM, Sigma), SR8278 (0.5 μM and 2 μM, Sigma), 3-(3-pyridinyl)-1-(4-pyridinyl)-2-propen-1-one (3PO, 25 μM, Calbiochem) or dehydroepiandrosterone (DHEA, 250 μM, Sigma) for 48 h.

### Cell synchronization

Once reaching 80–90% confluence, SGC-7901 cells were cultured with DMEM medium containing 50% FBS for 2 h for synchronization, which was considered as zeitgeber time 0 (ZT0)^17,18^. Cells were then kept in serum-free medium, and were harvested every 6 h. Total RNA was extracted for PFKFB3 and G6PD gene expression.

### Transfection

To reduce endogenous Rev-erbα and BMAL1 expression, cells were seeded onto a 12-well plate (5 × 10^5^/well) and transfected with human Rev-erbα or BMAL1 siRNA (Dharmacon RNA Technologies, Lafayette, CO, USA) at 50 nM for 24 h using the Lipofectamine reagent (Invitrogen, Carlsbad, CA, USA) according to the instructions. A RNA-guided CRISPR/Cas9-mediated genome editing approach^19^ was used to disrupt Rev-erbα gene in SGC-7901 cells. Rev-erbα targeting sgRNA (Santa Cruz, Cat#: SC-401211) was cloned into a lentiCRISPRv1 plasmid (Addgene, Cambridge, MA) to generate lentiCRISPR-Rev-erbα-sgRNA virus. A lentiCRISPRv1 plasmid that expressed an EGFP targeting sgRNA (Addgene) was used to generate control lentiCRISPR-EGFP-sgRNA virus, as previously described^20^. Cells were seeded in 6-well cell culture plates (1 × 10^5^ cells/well) in DMEM medium and transduced with lentiCRISPR-Rev-erbα-sgRNA virus or control lentiCRISPR-EGFP-sgRNA virus at the multiplicity of infection (MOI) of 0.3^20^. Cells were then selected in DMEM medium containing puromycin (1 µg/ml) for 2 weeks. Rev-erbα expression was determined by Western blot. Then Rev-erbα wild-type plasmids and plasmids lacking DNA binding domain (DBD)^21^ were transfected into Rev-erbα KO cells.

### Patients and tissues collection

All samples were obtained from 74 patients with diagnosed gastric cancer who were subjected to surgical operation at the First Affiliated Hospital of Anhui Medical University in 2014, as previously described^16,22^. The study population median age was 63.4 years (range: 33–84) and the gender distribution was 58 males and 16 females. The detailed clinical characteristics were described in our previous publications^16,22^. None of the patients had received any other therapies, such as radiotherapy or medical chemotherapy before surgery. All patients’ hepatic, renal and bone marrow function were normal. Eastern Cooperative Oncology Group (ECOG) performance status between 0 and 2. Patients were excluded with serious imbalance, pregnancy, and breast-feeding. Informed consent was obtained from all individual participants included in the study. The research protocol was approved by the Clinical Research Ethics Committee of Anhui Medical University. All methods were in accordance with the accurate guidelines to carry out.

### EdU-incorporation cell proliferation assay

Click-iT EdU Flow Cytometry kit was purchased from Thermo Fisher Scientific, Inc. (Waltham, MA, USA). Cell proliferation was performed according to the manufacturer’s instructions^23^. In brief, the cells were incubated with 5′-ethynyl-2′-deoxyuridine (EdU, 10 µM) for 2 h. Cells were harvested and mixed with 3 ml of PBS containing 1% bovine serum albumin (GE Healthcare Life Sciences), which was centrifuged at 1500 × *g* for 10 min at 4 °C and fixed with 100 µl 4% formaldehyde for 15 min. Cells were then washed and incubated with 100 µl saponin-based permeabilization buffer for 15 min. After permeabilization, the samples were incubated with CuSO4 and Alexa Flour® 488 coupled to azide for 30 min, and measured by the flow cytometry with 50,000 events.

### Glycolytic analysis

XF-24 Extracellular Flux Analyzer (Seahorse Bioscience) was used for real-time analysis of extracellular acidification rate (ECAR). Cells were seeded in Seahorse XF-24 cell culture microplates at the density of 20,000 cells/well. ECAR was recorded followed by sequential injections with 10 mM glucose, 1.0 μM oligomycin, and 50 mM 2-deoxy-D-glucose according to the manufacturer’s manual^24^.

### Lactate, glucose and NADPH measurement

Intracellular levels of lactate were determined using lactate assay kit (BioVision, Milpitas, CA) according to the manufacturer’s instructions. Glucose levels in the supernatants were determined using Glucose Colorimetric/Fluorometric Assay Kit (Biovision) according to the manufacturer’s instructions. Glucose consumption was calculated as fold change relative to untreated controls. Intracellular NADPH levels were determined using the NADP/NADPH Quantification Kit (Biovision) according to the manufacturer’s instructions.

### Immunohistochemistry

Rev-erbα, PFKFB3 and G6PD protein abundance in human gastric normal and cancer tissues was measured by immunohistochemistry^22^. Briefly, the specimens were blocked with 3% hydrogen peroxide, 10% normal goat serum for 10 min, respectively, and then incubated with their antibodies (1:100–1:250 dilutions, Abcam, USA) overnight at 4 °C. After incubation with biotin-conjugated secondary antibody (PV6000, ZSGB-BIO, China), the tissue slides were incubated with streptavidin-biotin horseradish peroxidase complex followed by incubation with diaminobenzidine (DAB, ZSGB-BIO, China) for 5 min. The counterstaining with hematoxylin was then performed, and the bright-field microscope was used to take images of stained samples in a single-blinded manner. The relative protein expression of all images was calculated in the mean optical density (MOD) units. The staining intensity was scored as ‘0’ (no staining), ‘1’ ( ≤ 25%, weakly stained), ‘2’ (25–50%, moderately stained), or ‘3’ ( ≥ 50%, strongly stained). A low REV-ERBα expression was defined as score ‘0’, ‘1’ or ‘2’, and a high REV-ERBα expression was defined as score ‘3’. The patients divided into two groups: low expression group (*n* = 43) and high expression group (*n* = 31) of Rev-erbα as we described^16^.

### Western blot

Cells were lysed in lysis buffer (25 mM HEPES, 2 mM MgCl2, 2 mM DTT, 1 mM EDTA, 1 mM PSMF, 5 µg/ml leupeptin, pH 7.4). Freeze-thawing the suspension liquid containing the extracted protein 3 times subsequently, the lysates were centrifuged at 10,000 rpm at 4 °C for 10 min^25^. The concentration of extracted protein in supernatants was determined by the BCA assay. The protein extracts (10–20 µg) were separated by 4–12% SDS–PAGE and transferred to polyvinylidene fluoride (PVDF) membranes. Following non-specific blocking with 5% skimmed milk at room temperature for 2 h, PVDF membrane was washed 3 times with TBST (TBS contained 0.1% Tween-20) for 10 min each time. The membranes were then incubated with antibodies against REV-ERBα (Cat#. ab174309; Abcam), PFKFB3 (Cat#: ab181861, Abcam) and G6PD (Cat#: ab993, Abcam) overnight at 4 °C, and then washed 3 times with TBST for 10 min each time. The membranes were incubated with the corresponding rabbit anti-goat horseradish peroxidase-conjugated secondary antibody (rabbit; cat no. AP106P; 1:5,000 dilution; EMD Millipore) for 2 h at 20 °C, after that washed 3 times with TBST again for 10 min each time. The detection of the molecules of interest was carried out using enhanced chemiluminescence (Beyotime Institute of Biotechnology, Haimen, China). The bands were quantified to calculate relative protein expression levels using Quantity one software version 4.99.5.2.0 (Bio-Rad Laboratories, Inc., Hercules, CA, USA).

### Reverse transcription quantitative polymerase chain reaction (RT-qPCR)

Total RNA was extracted from human tissues and cells using TRIzol® (Life Technologies; Thermo Fisher Scientific, Inc.). Total RNA was reverse transcribed by cDNA synthesis using a PrimeScript RT Reagent kit with gDNA Eraser (Perfect Real Time; Takara Bio, Inc., Otsu, Japan) at 37 °C for 30 min and 85 °C for 5 s. qPCR was operated using a 7900 Thermal Cycler (ABI, Applied Biosystems; Thermo Fisher Scientific, Inc.) with GoTaq® Green Master Mix (Promega Corporation, Madison, WI USA) at an initial denaturation at 95 °C for 30 s, followed by 40 cycles of denaturation for 5 s at 95 °C, annealing for 30 s at 60 °C and extension for 15 s at 72 °C. The qPCR primers were shown as follows: HKII, 5′-GGA CTG GAC CGT CTG AAT GT-3′ (forward) and 5′-ACA GTT CCT TCA CCG TCT GG-3′ (reverse); PFKL, 5′-GGC CGC GGT GGA CCT GGA GAA-3′ (forward) and 5′-TCA GAA GCC CTT GTC CAT GCT CAG G-3′ (reverse); PFKFB1, CCT GCA AAT CAG GAA GCA GT (forward) and TTT GCA AAC TGC AGG ATC AG (reverse); PFKFB2, TAC GAC TTC TTT CGG CAT GA (forward), and CTC CTC TCC CGG GTT GTA TT (reverse); PFKFB3, 5′-CAG TTG TGG CCT CCA ATA TC-3′ (forward) and 5′-GGC TTC ATA GCA ACT GAT CC-3′ (reverse); PKM, TCA CTC CAC AGA CCT CAT GG (forward), GAA GAT GCC ACG GTA CAG GT (reverse). The primers for β-actin were 5′-CAT GTA CGT TGC TAT CCA GGC-3′ (forward) and 5′-CTC CTT AAT GTC ACG CAC GAT-3 (reverse). The cycle threshold (Cq) values were obtained in each sample. Relative levels of mRNA were measured using the 2^-ΔΔCt^ method (10). β-actin was used as an internal gene for normalization.

### ChIP assay

Cells were fixed with 1% formaldehyde and terminated with 2.5 mM glycine. The scraped cells were sonicated for lysis in PBS with sodium thiosulfate. The lysates were divided into three aliquots, one of which was a positive control and received no treatment and one of which was a negative control, which was incubated with target protein. One-third of the cell lysate was served as the test group, and was incubated with antibody against MIST1 (1:100) and Protein G PLUS-Agarose. After removal of RNA and protein, DNA was extracted with phenol-chloroform, respectively. Next, the degree of enrichment on gene promoters was detected using real-time quantitative PCR.

### Statistical analyses

In vitro experiments were performed with at least three biological replicates based on our preliminary studies. Data are expressed as the mean ± standard deviation. Comparison between different groups was performed using analysis of variance. The Student-Newman-Keuls test was the post-hoc test used following analysis of variance. Data analysis was performed using SPSS 17.0 software (SPSS, Inc., Chicago, IL, USA). All reported *p* values were two-sided, and a value of *p* < 0.05 was considered statistically significant.

## Results

### Rev-erbα inhibits human gastric cancer cell proliferation

**Fig. 1:**
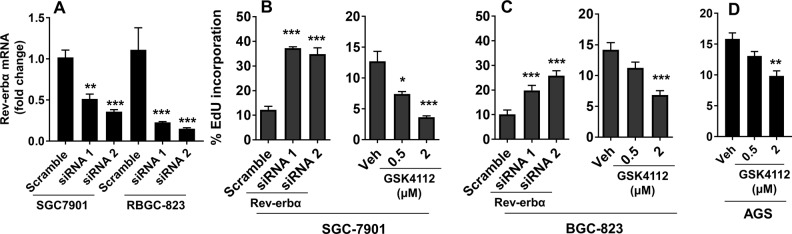
Rev-erbα suppresses proliferation of human gastric carcinoma cells. **a**–**c** SGC-7901 and BGC-823 cells were transfected with scramble and Rev-erbα siRNA for 48 h. GSK4112 (0.5 μM and 2 μM) was incubated for 48 h in SGC-7901 **b**, BGC-823 **c**, and AGC cells **d**. Cell proliferation was detected by flow cytometry using the Click-iT EdU cell proliferation assay kit. ^*^P < 0.05, ^***^P < 0.001 vs. Scramble siRNA or vehicle group, Mean ± SEM, *N* = 4–6

To determine the role of Rev-erbα on the proliferation in human gastric cancer cells, we transfected undifferentiated (BGC-823) and moderately differentiated (SGC-7901) gastric cancer cells with Rev-erbα siRNAs (Fig. 1a). As shown in Fig. 1b, c, knockdown of Rev-erbα gene increased the proliferation of both SGC-7901 and BGC-823 cells. Treatment with a Rev-erbα agonist GSK4112 (0.5 μM and 2 μM) for 48 h reduced the proliferation of SGC-7901, BGC-823, and AGS cells in a concentration-dependent manner. Compared to SGC-7901 cells, BGC-823, and AGS cells were not sensitive to GSK4112 (0.5 μM) to reduce cell proliferation (Fig. 1b–d). Altogether, Rev-erbα inhibits proliferation in human gastric cancer cells.

### Rev-erbα reduces glycolysis in human gastric cancer cells

**Fig. 2:**
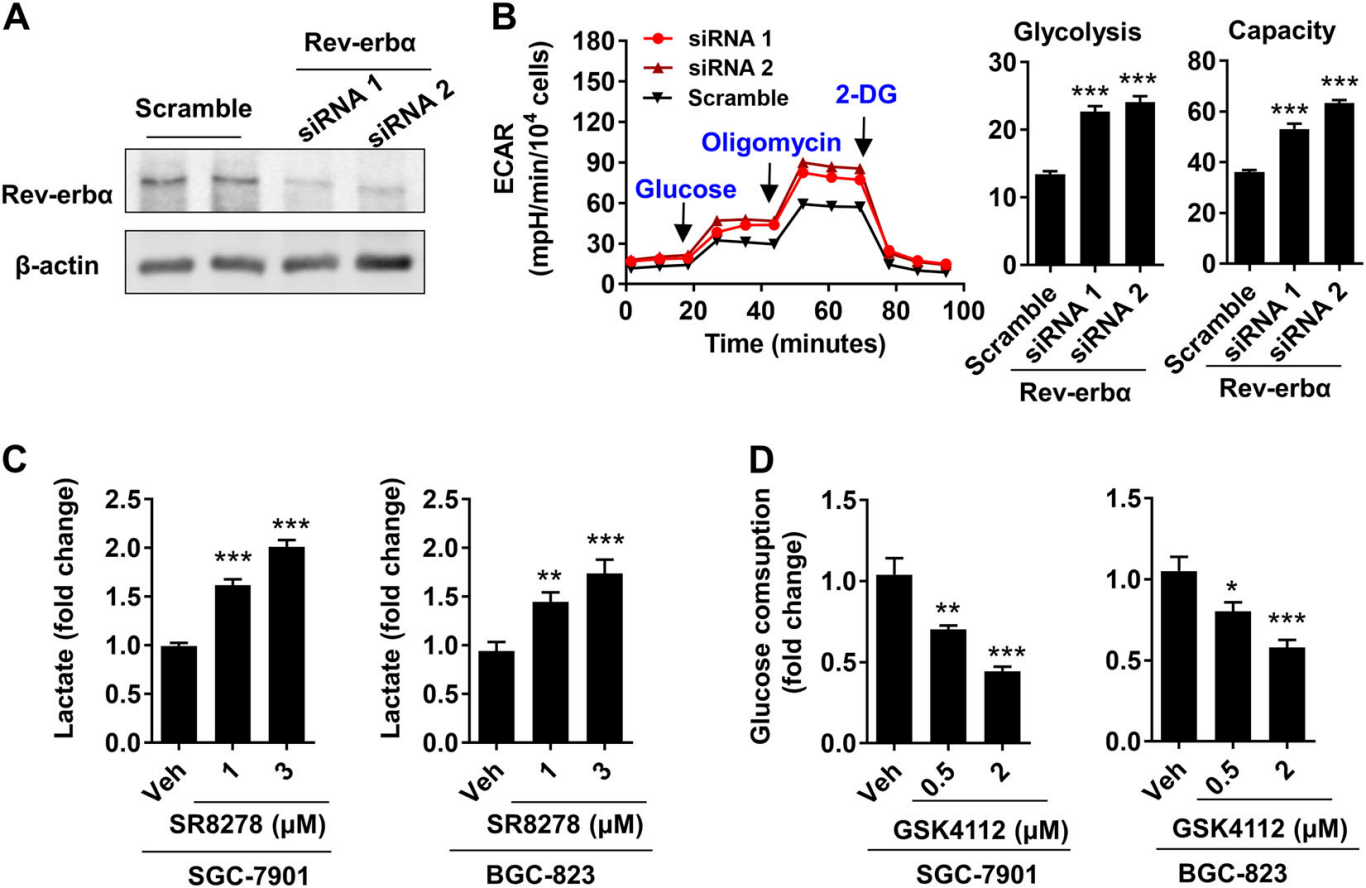
Rev-erbα inhibits glycolysis in human gastric carcinoma cells. **a** Rev-erbα siRNA was transfected into SGC-7901 cells for 48 h and Western blot was used to detect Rev-erbα protein levels. **b** The extracellular acidification rate (ECAR) of Rev-erbα siRNA transfected SGC-7901 was detected by Seahorse XF Analyzer, and normalized into cell number. **c** Both SGC-7901 and BGC-823 cells were treated with SR8278 (1–3 μM) for 48 h, and intracellular lactate was measured using a lactic acid test kit. **d** Cells were treated with GSK4112 (0.5–2 μM) for 48 h, and the glucose level in the cell supernatant was measured using a glucose test kit. ^*^P < 0.05, ^**^P < 0.01, ^***^P < 0.001 vs. Scramble siRNA or vehicle group. Mean ± SEM, *N* = 4–6

To determine the role of Rev-erbα on glycolysis in gastric cancer cells, we measured extracellular acidification rate (ECAR) using Seahorse XF Analyzer in SGC-7901 cells transfected with Rev-erbα siRNA. Transfection with Rev-erbα siRNA significantly reduced the protein levels of Rev-erbα (Fig. 2a). As shown in Fig. 2b, knocking down the Rev-erbα gene increased basal glycolysis and glycolytic capacity in SGC-7901 cells. Treatment with a Rev-erbα antagonist SR8278 (1 μM and 3 μM) for 48 h significantly increased intracellular levels of lactate in both SGC-7901 and BGC-823 (Fig. 2c). In contrast, treatment with a Rev-erbα agonist GSK4112 (0.5 μM and 2 μM, 48 h) reduced glucose consumption in both SGC-7901 and BGC-823 cells (Fig. 2d). Altogether, Rev-erbα reduces glycolysis in human gastric cancer cells.

### Rev-erbα inhibits the expression of genes encoding rate-limiting enzymes in glycolysis

To investigate whether Rev-erbα reduces glycolysis by inhibiting the gene encoding rate-limiting enzymes, we transfected Rev-erbα siRNA into SGC-7901 cells and detected the expression of HKII, PFK1, PFK2 (PFKFB1, PFKFB2, and PFKFB3 subtype), and PK using quantitative real-time PCR. As shown in Supplementary Fig. 1A, the mRNA levels of PFK1, PFKFB1, PFKFB2, or PK were not altered in SGC-7901 cells transfected with Rev-erbα siRNA. However, knockdown of Rev-erbα significantly increased the levels of HKII and PFKFB3 genes. Furthermore, chromatin immunoprecipitation was utilized to determine whether Rev-erbα is recruited on the promoters of these genes. It was found that Rev-erbα was recruited to the promoters of human PFKFB3 but not HKII gene (Supplementary Fig. 1B). These results demonstrate that Rev-erbα is recruited on the promoters of PFKFB3 gene, and suppresses its expression.

### Rev-erbα inhibits G6PD gene expression and NADPH generation

To investigate whether Rev-erbα regulates the PPP, we transfected Rev-erbα siRNA into SGC-7901 cells and detected the expression of G6PD gene using quantitative real-time PCR. As shown in Supplementary Fig. 2A, knockdown of Rev-erbα significantly increased the levels of G6PD gene. Furthermore, the levels of NADPH were reduced in SGC-7901 cells treated with GSK4112 (0.5 μM and 2 μM, 48 h) compared to vehicle-treated cells (Supplementary Fig. 2B). Chromatin immunoprecipitation was utilized to determine whether Rev-erbα is recruited on the promoters of this gene. It was found that Rev-erbα was recruited to the promoters of human G6PD gene (Supplementary Fig. 2C). These results demonstrate that Rev-erbα is recruited on the promoters of G6PD gene, and suppress the PPP.

### Rev-erbα modulates PFKFB3 and G6PD gene expression independent of BMAL1

It has been shown that PFKFB3 gene expression and G6PD enzymatic activity are rhythmic in certain cells and organs^26,27^. Thus, we determined the expression of PFKFB3 and G6PD genes at different zeitgeber times. The expression of both PFKFB3 and G6PD genes revealed the presence of multiple peaks every 12 h. Therefore, the expression of both PFKFB3 and G6PD was not circadian (approximately 24-hour cycles) in SGC-7901 cells (Supplementary Figure 3A, B).

Rev-erbα has been shown to regulate BMAL1 thereby controlling circadian rhythm^28^. Hence, we determined whether Rev-erbα modulates PFKFB3 and G6PD gene expression by modulating BMAL1. As shown in Supplementary Fig. 3C, BMAL1 gene expression was reduced in SGC-7901 cells when BMAL1 siRNA was transfected. As expected, GSK4112 treatment (2 μM) significantly reduced expression of PFKFB3 and G6PD genes in SGC-7901 cells (Supplementary Fig. 3D, E). However, knockdown of BMAL1 had no effects on PFKFB3 or G6PD gene expression in SGC-7901 cells treated with GSK4112 (2 μM) (Supplementary Fig. 3D and 13E). These results demonstrate that Rev-erbα modulates PFKFB3 and G6PD gene expression independent of BMAL1.

### Rev-erbα protein inhibits PFKFB3 and G6PD gene expression depending on its DNA binding domain (DBD)

To determine whether the Rev-erbα protein binds directly to the PFKFB3 and G6PD genes, the DBD of Rev-erbα protein was mutated so as to detect PFKFB3 and G6PD gene expression. First, we generated Rev-erbα KO SGC-7901 cells using the CRISPR/Cas9 system (Supplementary Fig. 4A). Next, we transfected the wild-type and mutant DBD plasmids of Rev-erbα in these KO cells, and determined PFKFB3 and G6PD mRNA levels. As shown in Supplementary Fig. 4B, mutation of Rev-erbα DBD significantly increased PFKFB3 and G6PD gene expression in SGC-7901 cells. Hence, Rev-erbα binds to PFKFB3 and G6PD DNA through its DBD.

### Rev-erbα inhibits gastric cancer cell proliferation by PFKFB3 and G6PD

**Fig. 3:**
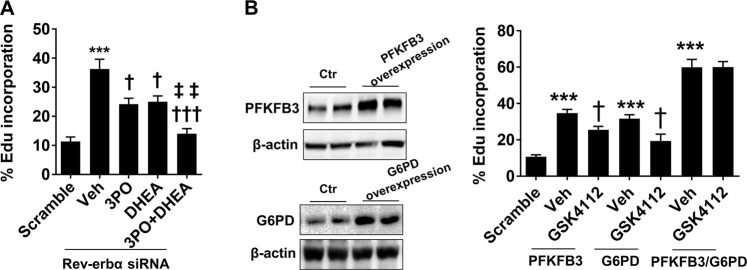
Rev-erbα inhibits proliferation of human gastric carcinoma cells by downstream PFKFB3 and G6PD signaling. EdU incorporation into DNA synthesis (cell proliferation) was detected using a Click-iT EdU cell proliferation assay kit. **a** SGC-7901 cells were transfected with scramble and Rev-erbα siRNA, and then treated with 3PO (25 μM) and DHEA (250 μM) for 48 h. **b** SGC-7901 cells were overexpressed with PFKFB3 and G6PD and treated with GSK4112 (2 μM) for 48 h. (Left panel) Western blot was used to detect PFKFB3 and G6PD protein levels. (Right panel) Cell proliferation assay. ^***^P < 0.001 vs. Scramble plasmids. ^†^*P* < 0.05, ^†††^*P* < 0.001 vs. siRNA/vehicle; ^‡‡^*P* < 0.01 vs. siRNA/3PO or siRNA/DHEA. Mean ± SEM, *N* = 4–5

Although Rev-erbα inhibited PFKFB3 and G6PD gene expression, it is unclear whether PFKFB3 and G6PD mediate the effect of Rev-erbα on proliferation of human gastric cancer cells. First, Rev-erbα siRNA was transfected into SGC-7901 cells and treated them with PFKFB3 and G6PD enzyme inhibitors 3-PO (25 μM) and DHEA (250 μM) for 48 h, respectively. Click-iT EdU cell proliferation assay and flow cytometry were used to detect the incorporation of EdU into DNA. As shown in Fig. 3a, both 3-PO and DHEA significantly reduced Rev-erbα knockdown-induced proliferation in SGC-7901 cells. Combined 3-PO and DHEA treatments further reduced proliferation induced by Rev-erbα knockdown in SGC-7901 cells compared to treatment alone. In contrast, overexpression of PFKFB3 and G6PD increased proliferation in SGC-7901 cells (Fig. 3b). Treatments with a Rev-erbα agonist GSK4112 (2 μM, 48 h) significantly reduced PFKFB3 and G6PD overexpression-induced proliferation in SGC-7901 cells (Fig. 3b). However, GSK4112 treatment did not affect the proliferation of SGC-7901 cells induced by dual overexpression of PFKFB3 and G6PD (Fig. 3b). These results suggest that Rev-erbα inhibits proliferation of human gastric cancer cells via downstream PFKFB3 and G6PD signaling.

### Glycolysis and the PPP are enhanced in patients with low expression of Rev-erbα in gastric cancer tissues

To extrapolate the in vitro findings into human samples, we determine the expression of PFKFB3 and G6PD as well as the metabolites, including lactate, pyruvate and NADPH in patients with gastric cancer. As shown in Supplementary Fig. 5A, the protein abundance of PFKFB3 and G6PD was increased in gastric cancer tissues, which was associated with increased TNM stage. This was confirmed by immunohistochemistry (Supplementary Fig. 5B).

**Fig. 4:**
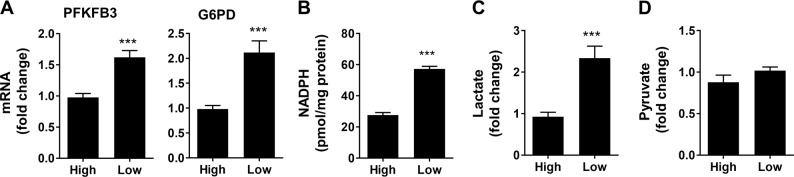
Glycolysis and the PPP are increased in human gastric cancer tissues with low Rev-erbα expression. G6PD and PFKFB3 mRNAs **a**, NADPH **b**, lactate **c**, and pyruvate **d** were measured in human gastric cancer tissues based on high and low Rev-erbα expression. Data are represented as the mean ± SD. *N* = 6–15. ***P < 0.001 versus group with high Rev-erbα expression

We next grouped the patients with gastric cancer into low and high expression of Rev-erbα, as we previously described^16^. We found that the mRNA levels of PFKFB3 and G6PD were augmented in patients with low abundance of Rev-erbα (Fig. 4a). Furthermore, the levels of lactate and NADPH were also increased in patients with low expression of Rev-erbα compared to the patients with high expression of Rev-erbα (Fig. 4b). The levels of pyruvate were not changed in patients with gastric cancer between low and high expression of Rev-erbα (Fig. 4c). Altogether, these results suggest that low expression of Rev-erbα associates with increased glycolysis and the PPP in gastric cancer.

## Discussion

We have shown that Rev-erbα was reduced in human gastric cancer, which was associated with poor differentiation, TMN stages and poor prognosis^16^. However, the mechanisms underlying these findings are not clear. In the present study, we found that Rev-erbα inhibits glycolysis and the PPP, thereby reducing proliferation in gastric cancer cells. This was due to increased expression of PFKFB3 and G6PD in human gastric cancer cells. Moreover, reduced Rev-erbα in patients with gastric cancer correlated with increased glycolysis and the PPP in cancer tissues. These findings provide potential therapeutic approaches by activating Rev-erbα to inhibit the proliferation of gastric cancer cells.

Rev-erbα is highly expressed in adipose tissue, skeletal muscle and brain, which plays important roles in lipid metabolism, adipocyte differentiation, inflammatory response, and circadian rhythm^28,29^. Although heme, a ligand of Rev-erbα, inhibits the expression of phosphoenolpyruvate carboxykinase involved in gluconeogenesis^30^, there are no reports regarding the role of Rev-erbα in glycolysis and the PPP. There are three rate-limiting enzymes, including HKII, PFK, and PK, in glycolysis and a rate-limiting enzyme G6PD in the PPP. We found that Rev-erbα could be recruited on the promoters of PFKFB3, an isoform of PFK, and G6PD genes, and directly inhibited their gene transcription in human gastric cancer cells. Along with the data on ECAR, lactate, and glucose consumption, Rev-erbα suppresses glycolytic flux and the PPP in gastric cancer cells by inhibiting PFKFB3 and G6PD gene expression. There are two pathways for Rev-erbα-mediated transcription repression. Once in nucleus, Rev-erbα competes with the retinoic acid-associated orphan receptor alpha (RORα) to bind with the ROR response element (RORE) of targeting genes, such as circadian gene Bmal1, inhibiting their transcription^28,31,32^. However, we found that knockdown of Bmal1 did not influence PFKFB3 or G6PD gene expression when cells were treated with GSK4112. These findings suggest that BMAL1 is not involved in Rev-erbα-mediated modulation on PFKFB3 or G6PD gene expression in gastric cancer cells. In the present study, we showed that PFKFB3 and G6PD genes did not exhibit rhythmic expression. This is not in agreement with the finding that the expression of these genes is rhythmic in tongue cancer cells^26,27^. This discrepancy may be due to cell-specific (gastric vs tongue cancer cells). The second pathway is that that Rev-erbα inhibits gene transcription by directly binding to target response elements (RevREs) and by recruiting the corepressor N-CoR and histone deacetylases on targeting gene promoters^33^. It remains elusive whether Rev-erbα inhibits PFKFB3 and G6PD gene expression using similar mechanisms in human gastric cancer cells.

It has been shown that Rev-erbα binds to the genome using both DBD-dependent and DBD-independent mechanisms^21^. We found that mutation of Rev-erbα DBD domain increased the mRNA slevels of PFKFB3 and G6PD in human gastric cancer cell. This suggest that Rev-erbα relies on its DBD, thereby inhibiting PFKFB3 and G6PD gene expression. Further study is required to determine on the mechanisms of increased HKII gene expression once Rev-erbα was knockdown, despite Rev-erbα was not recruited on HKII gene promoters.

The glycolysis and its parallel shunt PPP not only generate bioenergetics for meeting the demands of malignant cell growth, but also provide a variety of biosynthesis of macromolecules for their anabolism. This was corroborated by our findings that pharmacological inhibition of glycolysis and the PPP reduced proliferation in gastric cancer cells, whereas overexpression of PFKFB3 and G6PD augmented proliferation in these cells. In addition, inhibition of PFKFB3 and G6PD significantly attenuated Rev-erbα knockdown-induced increase in proliferation. These finding suggest that Rev-erbα inhibits proliferation in gastric cancer cells by suppressing PFKFB3 and G6PD gene expression. It is interesting to note that GSK4412-mediated inhibition of proliferation was abolished in dual PFKFB3 and G6PD overexpressing cells but not in individual overexpressing cells. This suggests that both PFKFB3 and G6PD are required for proliferation when Rev-erbα was reduced in human gastric cancer cells.

Utilizing clinical human samples, we found that the protein abundance of PFKFB3 and G6PD was increased in patients with gastric cancer. Furthermore, this was associated with increased TNM stage of human gastric cancer. This is corroborated by the findings that both PFKFB3 and G6PD are overexpressed in patients with gastric cancer, and promoted the proliferation and migration of gastric cancer cells^3,7^. It is interesting to note that the PPP and glycolysis were enhanced in human gastric cancer with low expression of Rev-erbα compared to gastric cancer tissues with high expression of Rev-erbα. These findings further conformed that Rev-erbα is reduced in gastric cancer, which leads to proliferation by promoting the PPP and glycolysis in gastric cancer cells.

In summary, we demonstrated for the first time that Rev-erbα was recruited to promoters of PFKFB3 and G6PD genes, thereby inhibiting glycolytic flux and the PPP, and subsequent proliferation in in vitro human gastric cancer cells. Furthermore, the PPP and glycolysis were enhanced in gastric cancer patients with low expression of Rev-erbα compared to gastric cancer tissues with high expression of Rev-erbα. Therefore, our findings provide a new biomarker and promising therapeutic strategy for the treatment of gastric carcinoma by targeting Rev-erbα-mediated metabolic reprogramming.

## Supplementary information

Supplemental Figure Legends

Supplemental Figure 1

Supplemental Figure 2

Supplemental Figure 3

Supplemental Figure 4

Supplemental Figure 5
