# Supplementary material for: Rev-erbα inhibits proliferation by reducing glycolytic flux and pentose phosphate pathway in human gastric cancer cells
Source: Oncogenesis. 2019 Oct 7;8(10):57. doi: 10.1038/s41389-019-0168-5 (PMC6779746; doi:10.1038/s41389-019-0168-5)
Supplement: Supplementary file 1 — Supplemental Figure Legends [file 41389_2019_168_MOESM1_ESM.doc]

**Rev-erbα inhibits proliferation by reducing glycolytic flux and pentose phosphate pathway in human gastric cancer cells**

Linlin Tao^1,2*^, Haoyuan Yu^1*^, Rui Liang^1^, Ru Jia^1^, Jinjin Wang^1^, Kai Jiang^1^, Zhengguang Wang**^1^**

^1^Department of General Surgery, First Affiliated Hospital of Anhui Medical University, Hefei, Anhui, People’s Republic of China; ^2^Division of Infectious Diseases, Department of Infectious Diseases, The First Affiliated Hospital of University of Science and Technology of China, Hefei, Anhui, People’s Republic of China

*These two authors contributed equally to this work

**Correspondence to:**

Zhengguang Wang, M.D., Ph.D,

Department of Surgery,

First Affiliated Hospital of Anhui Medical University,

Hefei 230032, Anhui, P.R.China.

Email: [wangzhengguang@ahmu.edu.cn](mailto:wangzhengguang@ahmu.edu.cn)

Tel: +86-13505699407

**Running title:** Re-erbα reduces glycolysis and pentose phosphate pathway

**SUPPLEMENTARY FIGURE LEGENDS**

**Supplementary Figure 1. Rev-erbα is recruited to the PFKFB3 gene promoter, thereby inhibiting their expression.** (A) Transfected Rev-erbα siRNA into SGC-7901 cells for 48 h, quantitative real-time PCR was performed to detect the expression of rate-limiting enzyme genes in glycolysis. (B) ChIP was performed to determine the recruitment of Rev-erbα on the promoters of HKII and PFKFB3 genes in both SGC-7901 and BGC-823 cells. ^***^P < 0.001 vs. Scramble siRNA (control, con). Mean ± SEM, N = 3-4.

**Supplementary Figure 2. Rev-erbα is recruited to G6PD gene promoter, thereby inhibiting its expression and NADPH generation.** (A) Transfected Rev-erbα siRNA into SGC-7901 cells for 48 h, quantitative real-time PCR was performed to detect the expression of G6PD gene. (B) SGC-7901 cells were treated with GSK4112 (0.5 μM and 2 μM) for 48 h, and NADPH was measured. (C) ChIP was performed to determine the recruitment of Rev-erbα on G6PD gene promoter. ^***^P < 0.001 vs. Scramble siRNA (control, con). Mean ± SEM, N = 3-4.

**Supplementary Figure 3. Rev-erbα regulates PFKFB3 and G6PD independent of BMAL1.** SGC-7901 cells were synchronized using serum shock, and the mRNAs of PFKFB3 (A) and G6PD (B) were measured at zeitgeber time 0 (ZT0), ZT6, ZT12, ZT18, ZT24, ZT30, ZT36, ZT42, and ZT48. (C-E) SGC-7901 were transfected with scramble and BMAL1 siRNA for 48 h, and then treated with GSK4112 (2 μM) for 48 h. The mRNAs of PFKFB3 (D) and G6PD (E) were measured by quantitative real-time PCR. ^*^P < 0.05, ^***^P < 0.001 vs. ZT0 or vehicle (Veh). Mean ± SEM, N = 5-6.

**Supplementary Figure 4. Mutation of Rev-erbα DBD increases PFKFB3 and G6PD gene expression.** (A) Transfection of Rev-erbα CRISPR/Cas9 KO plasmid into SGC-7901 cells. Western blot was used to detect Rev-erbα protein expression. (B) Schematic for Rev-erbα knockout SGC-7901 cells transfected with Rev-erbα wild type (WT) plasmids and plasmids lacking DBD (DBD mutant) using the floxp-cre system. (C) qPCR was performed to determine the expression of PFKFB3 and G6PD genes in Rev-erbα WT and DBD mutant cells. ^***^P < 0.001 vs. WT. Mean ± SEM, N = 3-4.

**Supplementary Figure 5. G6PD and PFKFB3 are increased in human gastric cancer tissues.** (A) Western blot was performed to determine the levels of G6PD and PFKFB3 proteins in in normal gastric tissues (Normal, N) as well as gastric cancer tissues from patients with TNM I, III, and IV stages. β-actin was used as housekeeping control for normalization. (B) Immunohistochemistry was performed to detect the abundance of G6PD and PFKFB3 proteins in normal gastric tissues (Normal, N) as well as gastric cancer tissues from patients with TNM III, and IV stages. Scale bars = 100 μm. Data are represented as the mean ± SD. N=5. **P*<0.05, ***P*<0.01, ****P*<0.001 versus Normal gastric tissues.
