# Supplementary figures and images for: Rev-erbα inhibits proliferation by reducing glycolytic flux and pentose phosphate pathway in human gastric cancer cells
Source: Oncogenesis. 2019 Oct 7;8(10):57. doi: 10.1038/s41389-019-0168-5 (PMC6779746; doi:10.1038/s41389-019-0168-5)

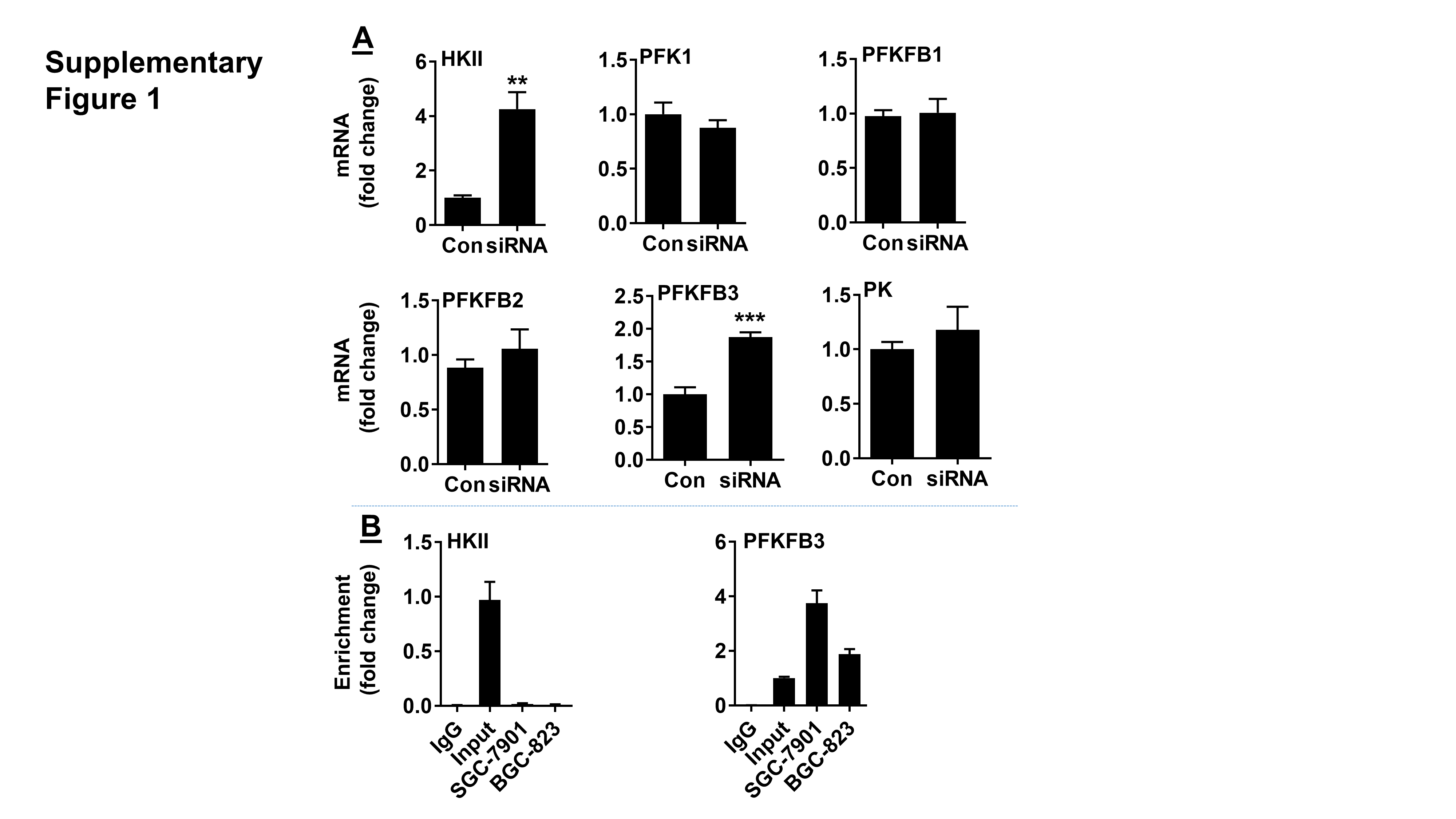

Supplement: Supplementary file 2 — Supplemental Figure 1 [file 41389_2019_168_MOESM2_ESM.tif]

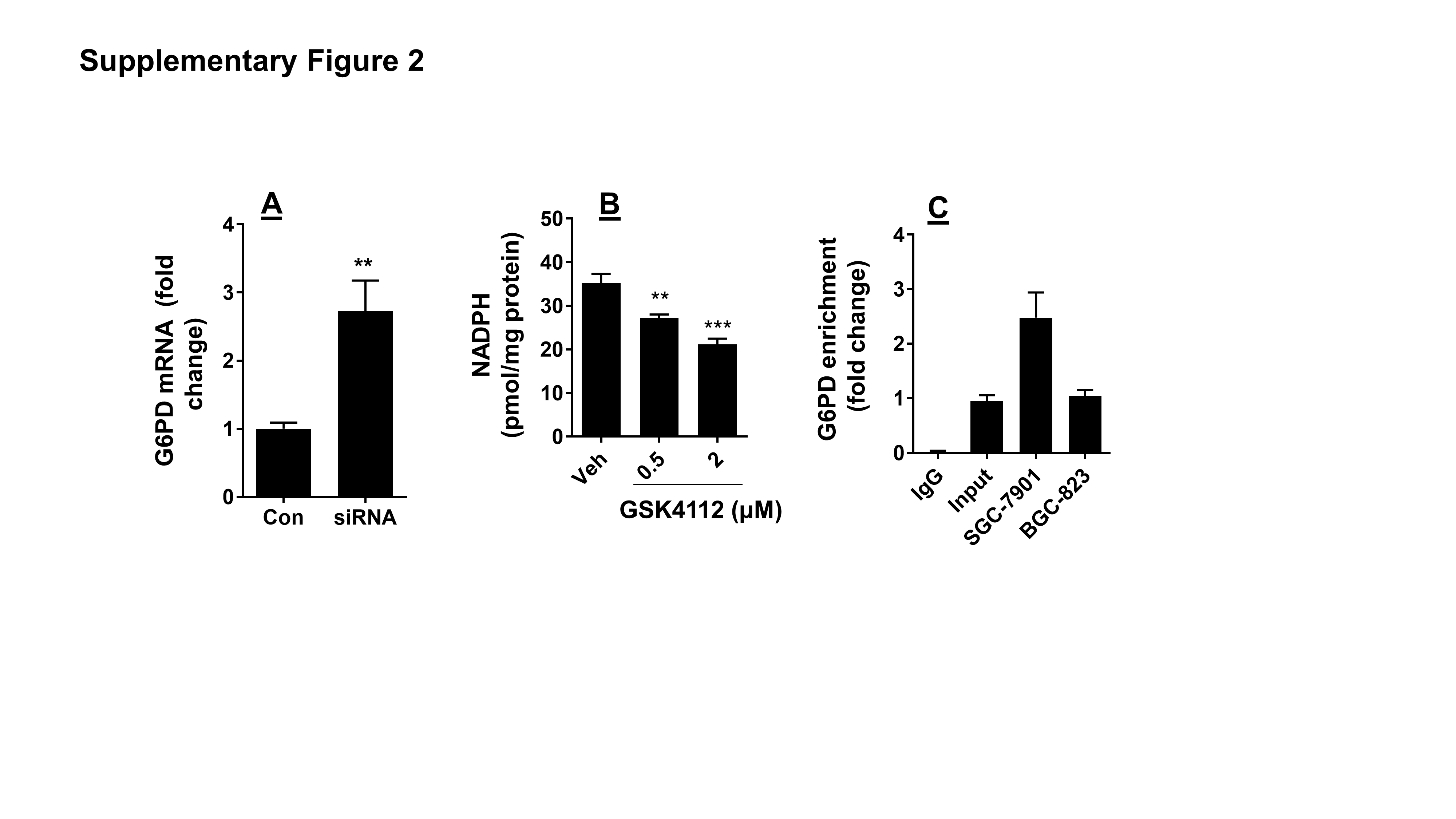

Supplement: Supplementary file 3 — Supplemental Figure 2 [file 41389_2019_168_MOESM3_ESM.tif]

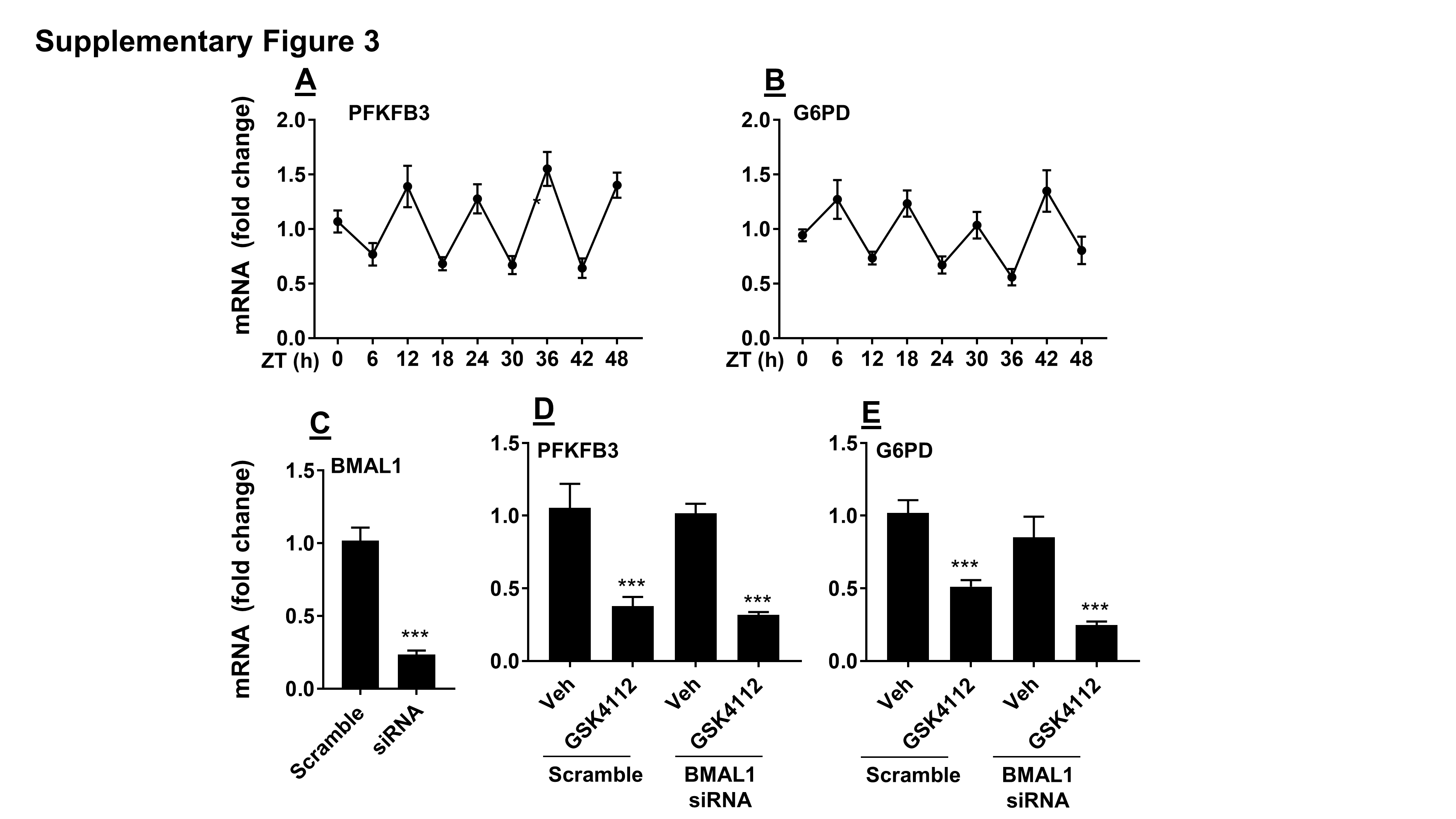

Supplement: Supplementary file 4 — Supplemental Figure 3 [file 41389_2019_168_MOESM4_ESM.tif]

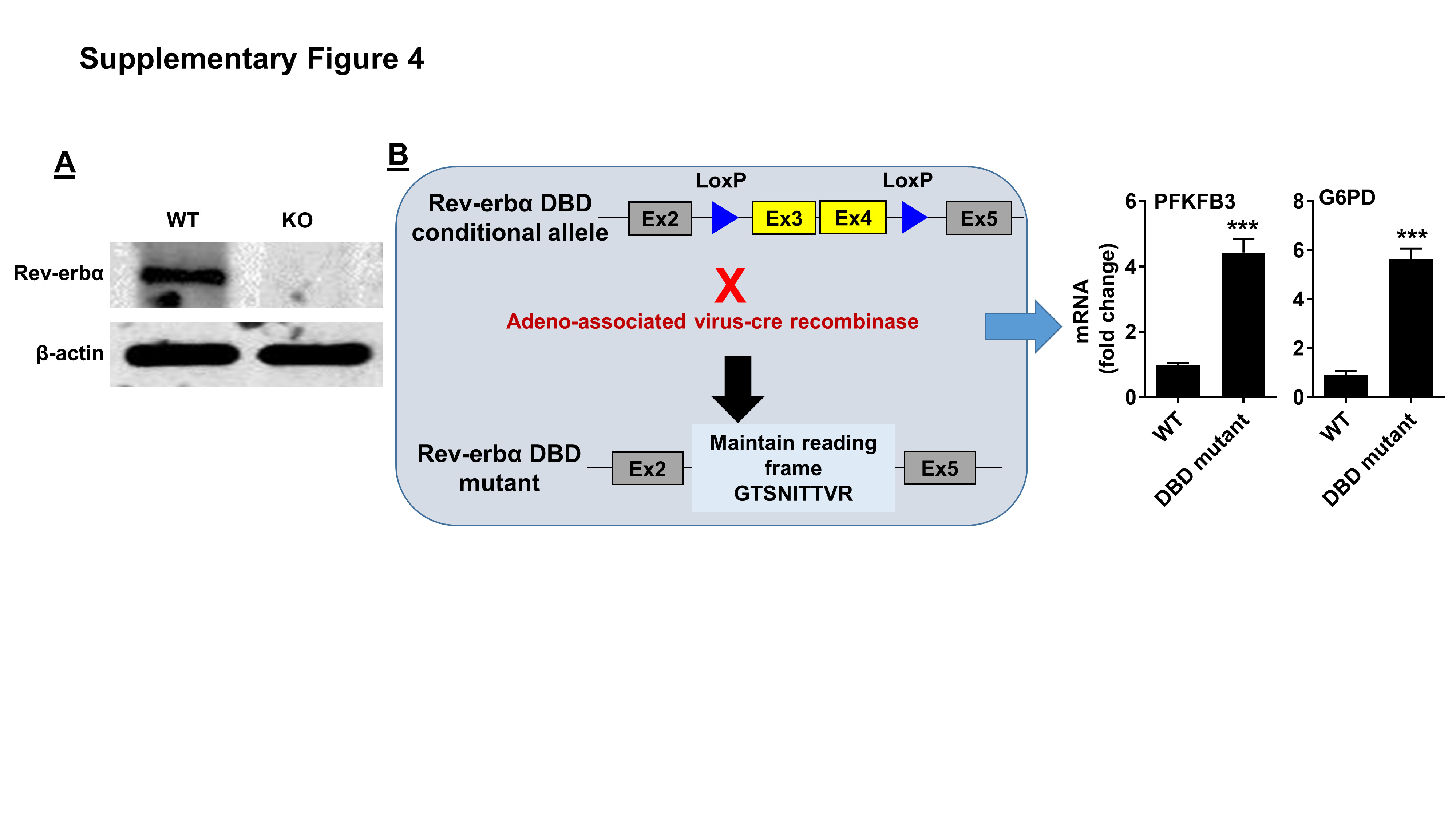

Supplement: Supplementary file 5 — Supplemental Figure 4 [file 41389_2019_168_MOESM5_ESM.tif]

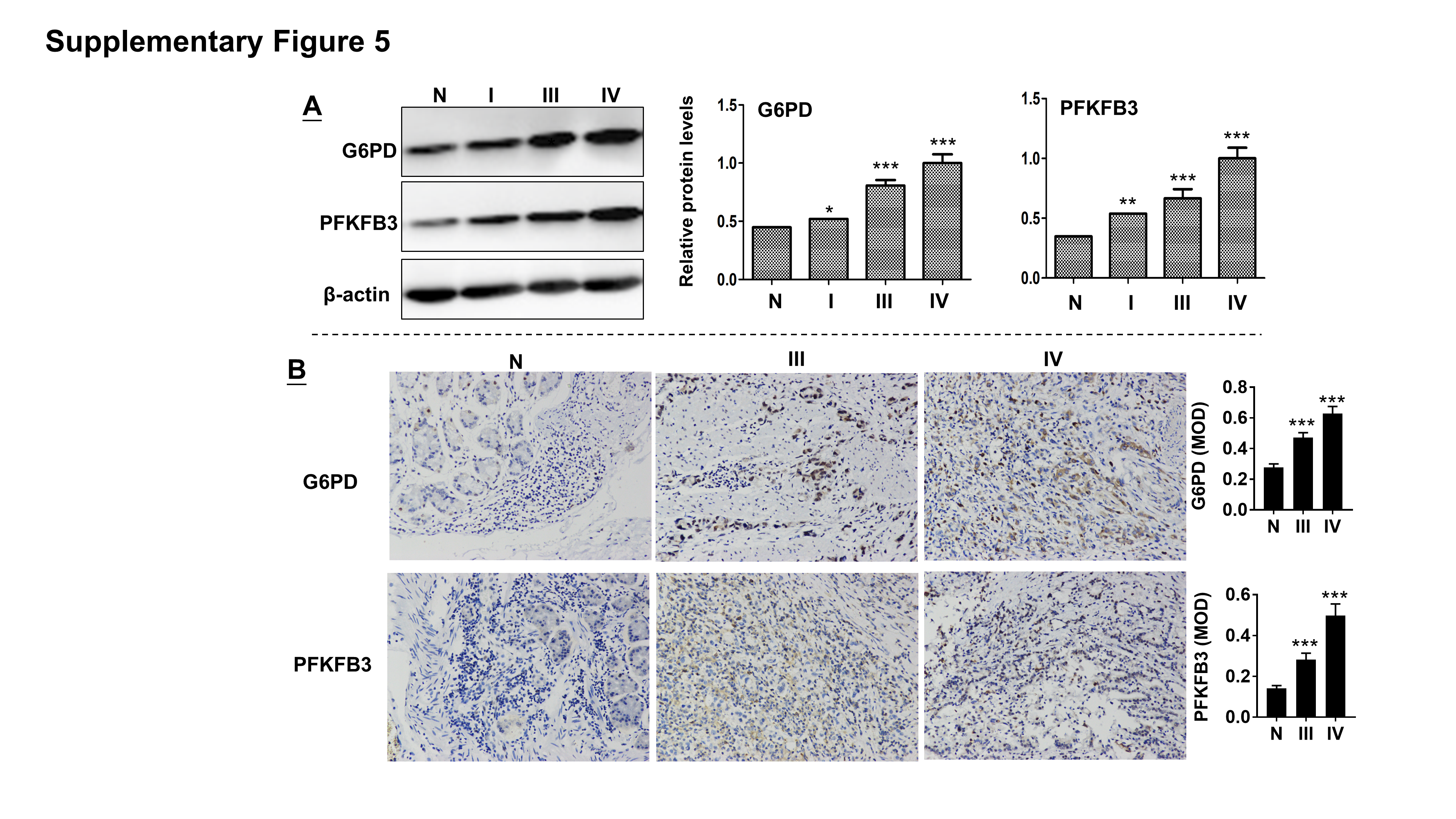

Supplement: Supplementary file 6 — Supplemental Figure 5 [file 41389_2019_168_MOESM6_ESM.tif]
